# Supplementary material for: Structural and Proteomic Changes in Viable but Non-culturable Vibrio cholerae
Source: Front Microbiol. 2019 Apr 17;10:793. doi: 10.3389/fmicb.2019.00793 (PMC6479200; doi:10.3389/fmicb.2019.00793)
Supplement: Supplementary file 12 [file Data_Sheet_1.PDF]

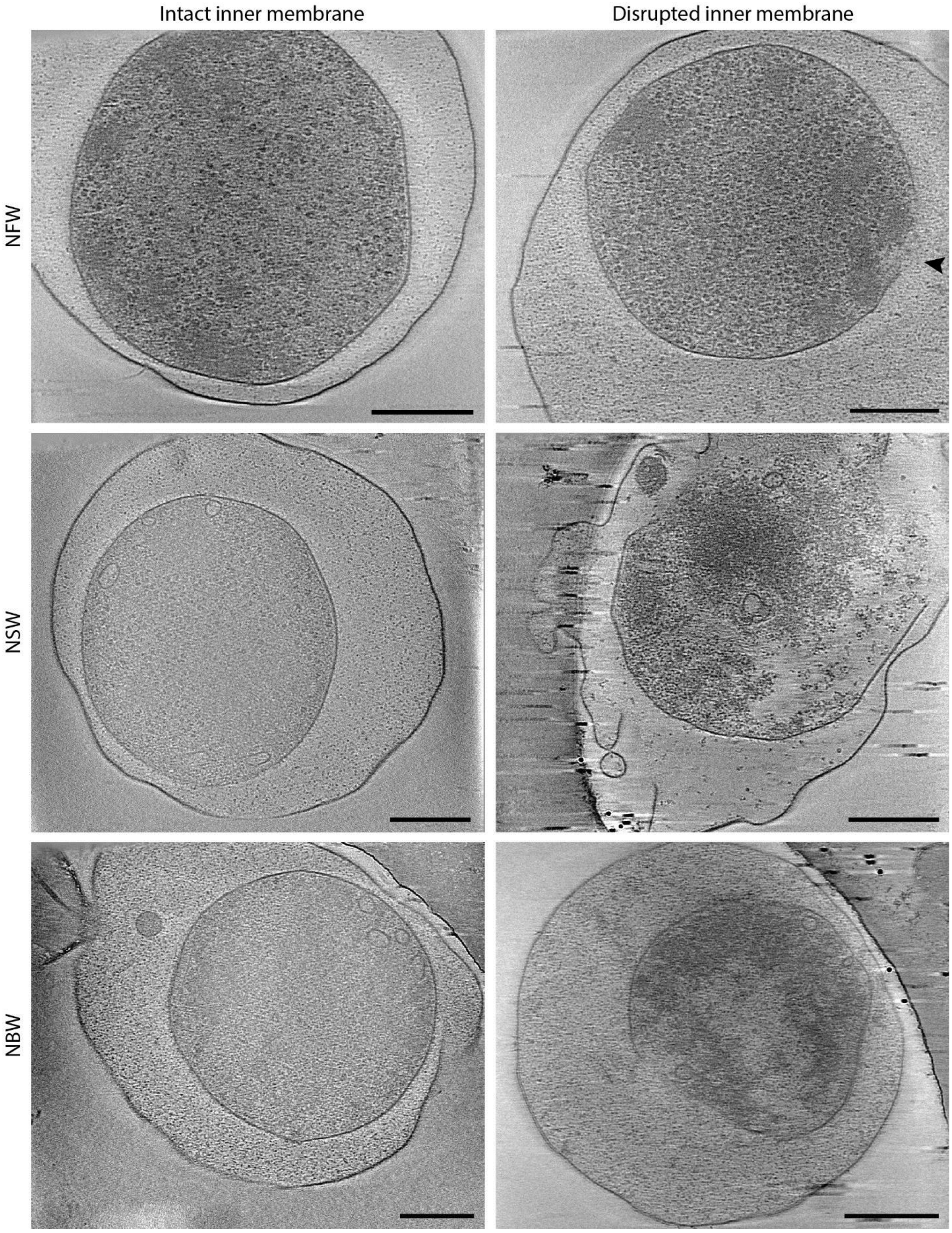

Figure S1: Exemplary intact (left row) and lyzed (right row) VBNC cells from NFW, NSW and NBW microcosms. Black arrow points to the lesion in the cytoplasmic membrane of the VBNC cell from a NFW microcosm. Scale bars represent 200 nm.

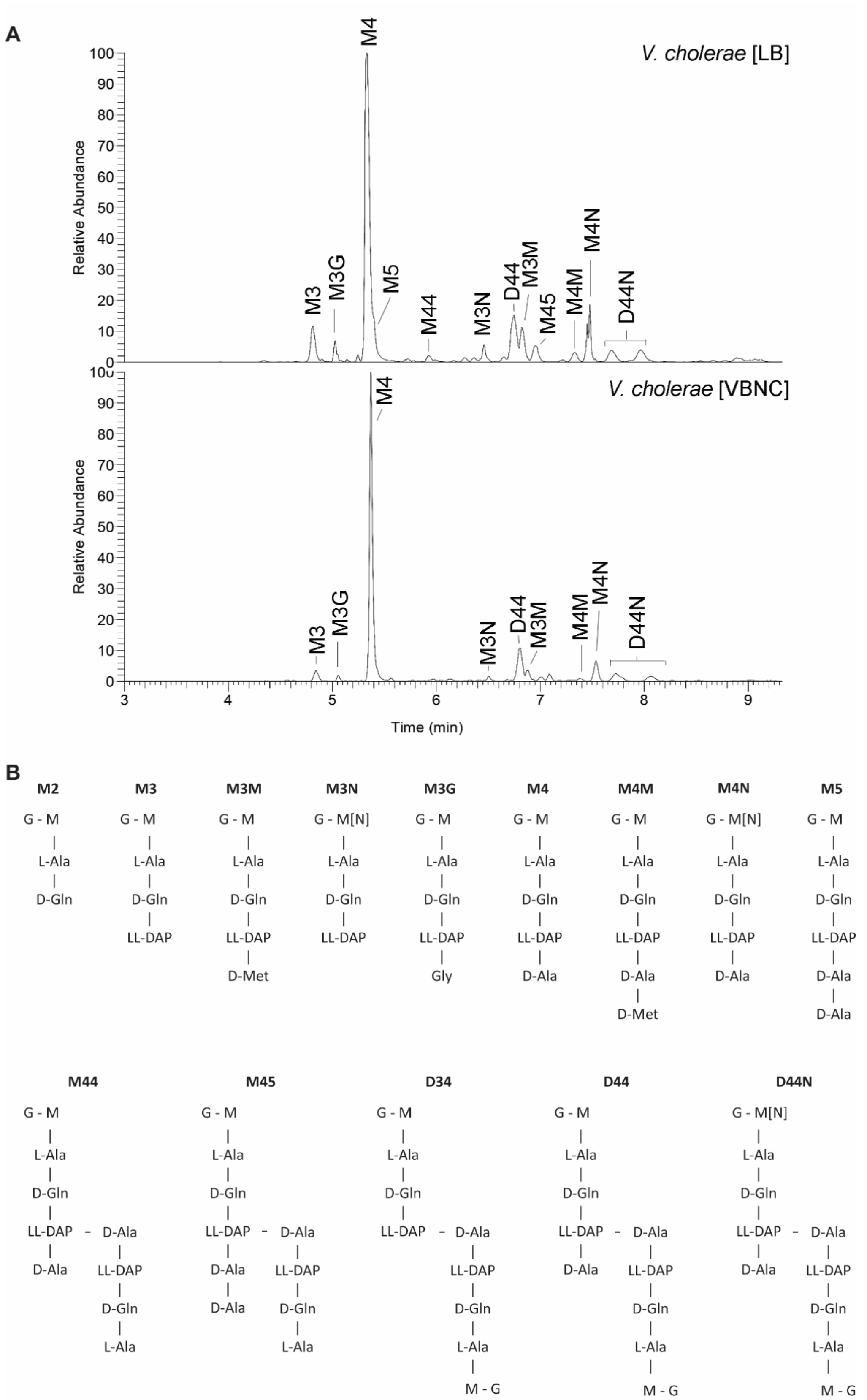

Figure S2: LC-MS analysis of *V. cholerae* from LB ON cultures (A, upper panel) and VBNC NSW cells (A, lower panel). Total Ion Current (TIC) with the relative abundance in arbitrary units (au) are depicted, peak designations can be inferred from table S1. Illustrations of structures of main monomers and dimers observed in the PG analysis are shown in B.

Table S1: Peptidoglycan composition in *V. cholerae* from LB ON cultures and NSW VBNC microcosms. Theoretical and observed masses are listed as m/z. Relative abundance of muropeptides are listed in percent. rt = retention time. Monomers and dimers are shown as separate sets, since dimers and monomers seem to be ionized differently by the MS.

Table S2: Unannotated proteomic data.

Table S3: Annotated proteomic data. Protein functions were inferred from Uniprot (UniProt Consortium, 2018), localization was predicted by PSORTdb (Peabody et al., 2016) and Fur binding boxes were inferred from (Mey et al., 2005; Panina et al., 2001). Data from a microarray study (Asakura et al., 2007) and RNA sequencing analysis (Xu et al., 2018) of *V. cholerae* VBNC cells are included for comparison.

Table S4: Functional analysis of all significantly (q<0.01) more or less abundant proteins in VBNC cells compared to cells from LB ON cultures. Enriched GO term clusters deducted using DAVID (Huang et al., 2009b, 2009a). Count lists the number of proteins associated with the respective term.

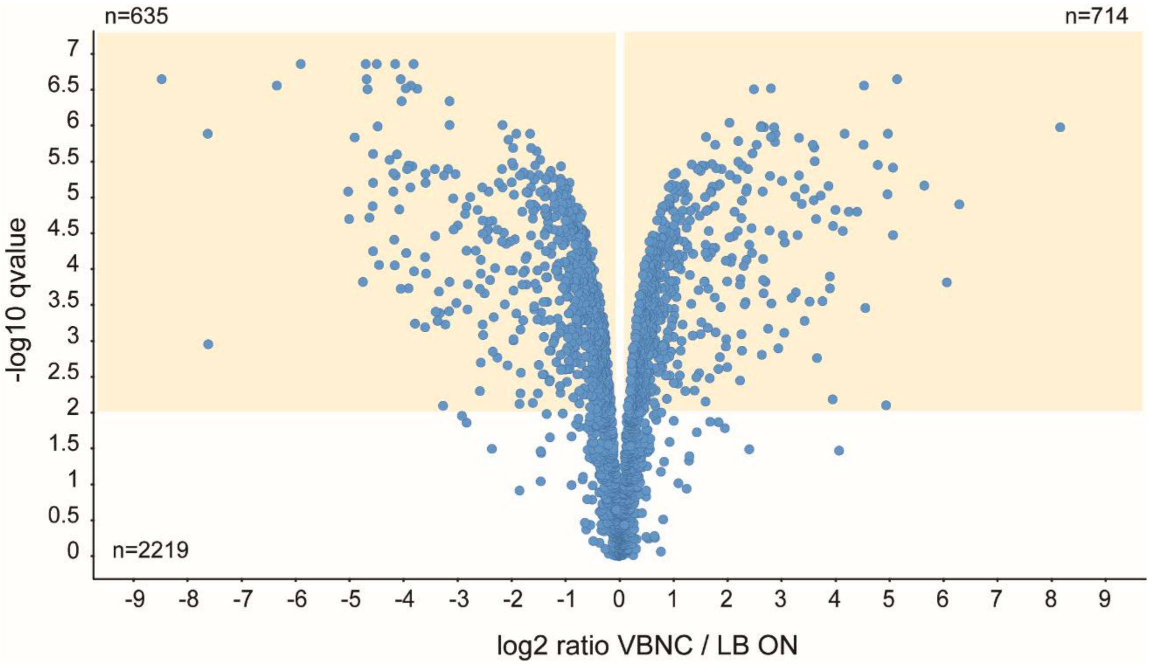

Figure S3: Comparative proteome analysis of the *V. cholerae* LB ON reference and VBNC culture. The distribution of proteome data is shown by plotting the fold change (log2 ratio VBNC/LB ON) of intensities between the *V. cholerae* VBNC cells and cells from LB ON cultures versus the statistical significance (−log10 q-value). The significance area is color coded (-log qvalue >=2).

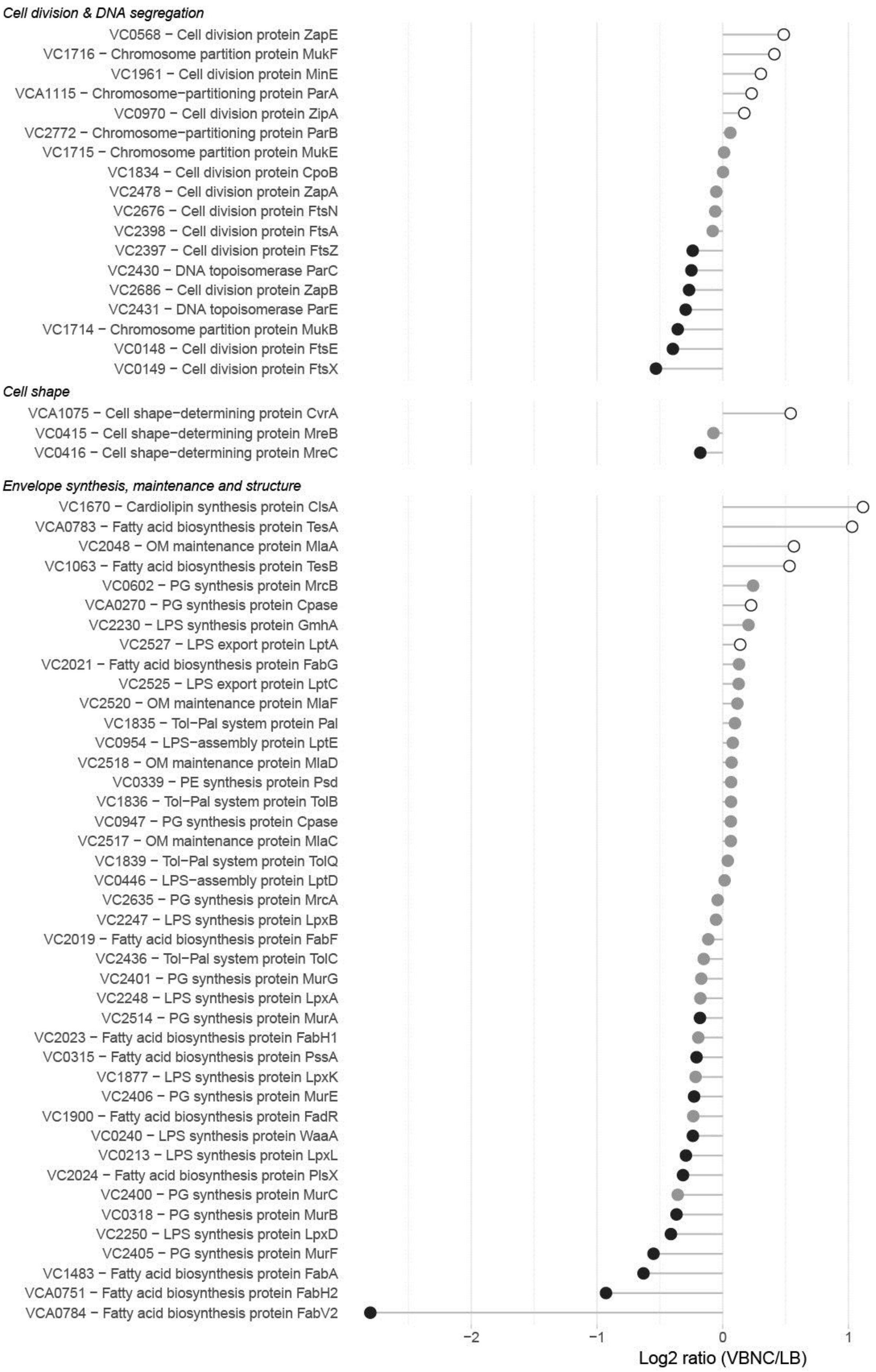
Cell division & DNA segregation
VC0568 − Cell division protein ZapE
VC1716 − Chromosome partition protein MukF
VC1961 − Cell division protein MinE
VCA1115 − Chromosome−partitioning protein ParA
VC0970 − Cell division protein ZipA
VC2772 − Chromosome−partitioning protein ParB
VC1715 − Chromosome partition protein MukE
VC1834 − Cell division protein CpoB
VC2478 − Cell division protein ZapA
VC2676 − Cell division protein FtsN
VC2398 − Cell division protein FtsA
VC2397 − Cell division protein FtsZ
VC2430 − DNA topoisomerase ParC
VC2686 − Cell division protein ZapB
VC2431 − DNA topoisomerase ParE
VC1714 − Chromosome partition protein MukB
VC0148 − Cell division protein FtsE
VC0149 − Cell division protein FtsX
Cell shape
VCA1075 − Cell shape−determining protein CvrA
VC0415 − Cell shape−determining protein MreB
VC0416 − Cell shape−determining protein MreC
Envelope synthesis, maintenance and structure
VC1670 − Cardiolipin synthesis protein ClsA
VCA0783 − Fatty acid biosynthesis protein TesA
VC2048 − OM maintenance protein MlaA
VC1063 − Fatty acid biosynthesis protein TesB
VC0602 − PG synthesis protein MrcB
VCA0270 − PG synthesis protein Cpase
VC2230 − LPS synthesis protein GmhA
VC2527 − LPS export protein LptA
VC2021 − Fatty acid biosynthesis protein FabG
VC2525 − LPS export protein LptC
VC2520 − OM maintenance protein MlaF
VC1835 − Tol−Pal system protein Pal
VC0954 − LPS−assembly protein LptE
VC2518 − OM maintenance protein MlaD
VC0339 − PE synthesis protein Psd
VC1836 − Tol−Pal system protein TolB
VC0947 − PG synthesis protein Cpase
VC2517 − OM maintenance protein MlaC
VC1839 − Tol−Pal system protein TolQ
VC0446 − LPS−assembly protein LptD
VC2635 − PG synthesis protein MrcA
VC2247 − LPS synthesis protein LpxB
VC2019 − Fatty acid biosynthesis protein FabF
VC2436 − Tol−Pal system protein TolC
VC2401 − PG synthesis protein MurG
VC2248 − LPS synthesis protein LpxA
VC2514 − PG synthesis protein MurA
VC2023 − Fatty acid biosynthesis protein FabH1
VC0315 − Fatty acid biosynthesis protein PssA
VC1877 − LPS synthesis protein LpxK
VC2406 − PG synthesis protein MurE
VC1900 − Fatty acid biosynthesis protein FadR
VC0240 − LPS synthesis protein WaaA
VC0213 − LPS synthesis protein LpxL
VC2024 − Fatty acid biosynthesis protein PlsX
VC2400 − PG synthesis protein MurC
VC0318 − PG synthesis protein MurB
VC2250 − LPS synthesis protein LpxD
VC2405 − PG synthesis protein MurF
VC1483 − Fatty acid biosynthesis protein FabA
VCA0751 − Fatty acid biosynthesis protein FabH2
VCA0784 − Fatty acid biosynthesis protein FabV2
−2
−1
0
1
Log2 ratio (VBNC/LB)

Figure S4: Relative abundances of proteins involved in cell division, determining cell shape, and cell envelope synthesis and maintenance. Depicted is the $\log_2$-ratio of NBW VBNC cells and LB ON cells. Proteins found in significantly higher abundance in VBNC cells compared to LB ON are indicated by open white circles while significantly lower ratios are shown as black circles. Protein abundances that were not significantly different are colored grey ($q<0.01$).

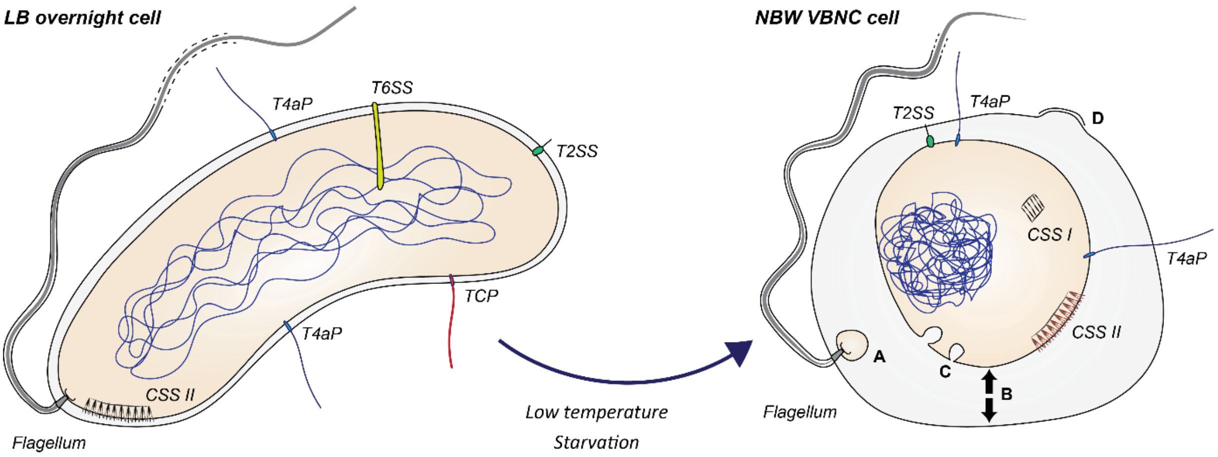

Figure S5: Schematic illustration of observed structural and morphological changes in *V. cholerae* VBNC cells from NBW microcosms. Cells from LB overnight cultures are rod-shaped and produce multiple different large protein structures such as the chemosensory cluster II array (CSS II), the flagellum, type IV a pili (T4aP), the TCP pilus (TCP), type VI secretion systems (T6SS) and type II secretion systems (T2SS). The outer and inner membrane are at a defined distance. Upon prolonged starvation in NBW at 4°C, cells lose their shape, the cytoplasm may be split into two compartments of different size (A) and the membranes are spaced further apart (B). The inner membrane forms invaginations (C) while the outer membrane presents several convex patches (D). Envelope spanning structures such as secretion systems and pili are likely not properly embedded. The chromosomal DNA appears condensed. Chemosensory system I arrays (CSS I) are observed while the MCPs of CSS II are present in changed abundances.

Table S5: Proteomic data of predicted metabolic pathways mapped using the Kyoto Encyclopedia of Genes and Genomes (KEGG) database (Kanehisa et al., 2017; Kanehisa and Goto, 2000).

Table S6: Proteomic data of predicted regulons as inferred from RegPrecise 3.0 (Novichkov et al., 2013).

Table S7: Relative abundances of predicted ABC transport systems. Components predicted to be in the same transport systems were grouped together. Proteins are only included if their peptides is $n>2$ and $q<0.01$.

References

Asakura, H., Ishiwa, A., Arakawa, E., Makino, S. I., Okada, Y., Yamamoto, S., et al. (2007). Gene expression profile of Vibrio cholerae in the cold stress-induced viable but non-culturable state. *Environ. Microbiol.* 9, 869–879. doi:10.1111/j.1462-2920.2006.01206.x.

Huang, D. W., Sherman, B. T., and Lempicki, R. A. (2009a). Bioinformatics enrichment tools: Paths toward the comprehensive functional analysis of large gene lists. *Nucleic Acids Res.* 37, 1–13. doi:10.1093/nar/gkn923.

Huang, D. W., Sherman, B. T., and Lempicki, R. A. (2009b). Systematic and integrative analysis of large gene lists using DAVID bioinformatics resources. *Nat. Protoc.* 4, 44–57. doi:10.1038/nprot.2008.211.

Kanehisa, M., Furumichi, M., Tanabe, M., Sato, Y., and Morishima, K. (2017). KEGG: new perspectives on genomes, pathways,diseases and drugs. *Nucleic Acids Res.* 45, 1–15. doi:10.1093/nar/gkw1092.

Kanehisa, M., and Goto, S. (2000). KEGG: Kyoto Encyclopaedia of Genes and Genomes. *Nucl. Acids Res.* 28, 27–30. doi:10.1093/nar/28.1.27.

Mey, A. R., Wyckoff, E. E., Kanukurthy, V., Fisher, C. R., and Payne, S. M. (2005). Iron and Fur regulation in Vibrio cholerae and the role of Fur in virulence. *Infect. Immun.* 73, 8167–8178. doi:10.1128/IAI.73.12.8167-8178.2005.

Novichkov, P. S., Kazakov, A. E., Ravcheev, D. A., Leyn, S. A., Kovaleva, G. Y., Sutormin, R. A., et al. (2013). RegPrecise 3.0 - A resource for genome-scale exploration of transcriptional regulation in bacteria. *BMC Genomics* 14. doi:10.1186/1471-2164-14-745.

Panina, E. M., Mironov, A. A., and Gelfand, M. S. (2001). Comparative analysis of FUR regulons in gamma-proteobacteria. *Nucleic Acids Res.* 29, 5195–5206. doi:10.1093/nar/29.24.5195.

Peabody, M. A., Laird, M. R., Vlasschaert, C., Lo, R., and Brinkman, F. S. L. (2016). PSORTdb: Expanding the bacteria and archaea protein subcellular localization database to better reflect diversity in cell envelope structures. *Nucleic Acids Res.* doi:10.1093/nar/gkv1271.

UniProt Consortium, T. (2018). UniProt: the universal protein knowledgebase. *Nucleic Acids Res.* 46, 2699–2699. doi:10.1093/nar/gky092.

Xu, T., Cao, H., Zhu, W., Wang, M., Du, Y., Yin, Z., et al. (2018). RNA-seq-based monitoring of gene expression changes of viable but non-culturable state of *Vibrio cholerae* induced by cold seawater. *Environ. Microbiol. Rep.* doi:10.1111/1758-2229.12685.
